# Complete Genome Sequence of Xanthomonas arboricola pv. *pruni* Strain Xcp1 Isolated in 1984 from a Bacterial Spot Spring Canker on Prunus persica var. *nucipersica* cv. “Redgold”

**DOI:** 10.1128/mra.00209-22

**Published:** 2022-11-09

**Authors:** Katherine M. D’Amico-Willman, Prasanna Joglekar, Emily K. Luna, David F. Ritchie, Jennie Fagen, Alejandra I. Huerta

**Affiliations:** a Department of Entomology and Plant Pathology, North Carolina State University, Raleigh, North Carolina, USA; b Department of Agricultural Biology, Colorado State University, Fort Collins, Colorado, USA; University of Maryland School of Medicine

## Abstract

Xanthomonas arboricola pv. *pruni* is an important plant pathogen and the causal agent of bacterial spot of stone fruits (*Prunus* spp). Here, we report a complete genome of X. arboricola pv. *pruni* strain Xcp1 generated from hybrid PacBio Sequel and Illumina NextSeq2000 sequencing.

## ANNOUNCEMENT

Xanthomonas arboricola pv. *pruni* is the causal agent of bacterial spot of *Prunus* spp., a destructive and widespread disease that affects peaches (1, 2). Several X. arboricola pv. *pruni* strains have been isolated and described; however, genomic resources are limited (2, 3).

Xcp1 was originally isolated from a spring canker on nectarine (Prunus persica var. *nucipersica*) cv. “Redgold” in April 1984 from a symptomatic tree canker at the North Carolina Sandhills Research Station (35.185679, −79.677848; Jackson Springs, NC). A section of the canker was excised using a sterile scalpel, macerated in sterile water, and streaked onto solid sucrose peptone agar (SPA). After 72 h at 28°C, a single colony was selected and purified.

Xcp1 was grown on solid SPA with no antibiotics for two days. DNA was isolated using the Wizard genomic DNA purification kit (Promega, Madison, WI, USA) following the manufacturer’s instructions and quantified using NanoDrop One (ThermoFisher Scientific, Waltham, MA, USA). Libraries were prepared at the Microbial Genome Sequencing Center (Pittsburgh, PA, USA) using the Illumina DNA Prep kit (Illumina, Inc., San Diego, CA, USA) following the manufacturer’s instructions and sequenced on the Illumina NextSeq 2000 platform (2 × 151 bp), generating 1,706,564 reads. Demultiplexing, quality control, and adapter trimming were performed with bcl-convert v.3.9.3 (4).

Xcp1 grown on solid nutrient agar as described above was used for PacBio sequencing. High-molecular-weight DNA was isolated using phenol-chloroform-isoamyl alcohol (25:24:1) and resuspended in 10 mM Tris-HCl containing RNase A (0.1 mg/mL) (5). Libraries were prepared following the PacBio protocol “Preparing multiplexed microbial libraries using SMRTbell Express template prep kit 2.0.” DNA was sheared to 10 kB with no size selection and was sequenced on the Sequel system (v.6.0 Sequel chemistry), generating 143,980 reads. Base calling and adapter trimming were performed by the Sequel system instrument control software v.6.0.0.46934.

The genome was assembled, rotated and trimmed, and circularized with the Trycycler (6) long-read consensus assembler using default parameters. Briefly, long reads were assembled with Flye, miniasm and minipolish, and Raven (7–9). The resulting assembly was polished with Illumina reads using polypolish (10) and POLCA (11). The final assembly contained a circular, 5,113,502-bp chromosome (187× coverage) and a 41,080-bp plasmid (560× coverage) with GC contents of 65.38 and 62.32%, respectively (Table 1). The assembly had a 99.95% average nucleotide identity (ANI) (12) to the X. arboricola pv. *pruni* reference genome contigs NZ_CP091075.1 and NZ_CP091076.1.

**TABLE 1:** Description of the whole-genome sequence of the Xcp1 chromosome and plasmid

Sequence type	Accession no.	Size (bp)	Coverage (×)	% GC	No. of^*a*^:			
					CDSs	rRNA operons	tRNAs	ncRNAs
Chromosome	CP090954.2	5,113,502	187	65.38	4,291	2	53	37
Plasmid	CP096275.1	41,080	560	62.32	48	0	0	0

a CDSs, coding DNA sequences; ncRNAs, noncoding RNAs.

The genome was annotated using the NCBI Prokaryotic Genome Annotation Pipeline (PGAP) (13–15) (Table 1). Conserved marker gene analysis using CheckM v.1.0.18 suggested the genome was 99.91% complete with 0% contamination (16). Default parameters were used for all software.

### Data availability.

Sequences have been submitted to GenBank under the following accession numbers: genome, CP090954.2; raw reads, CP096275.1; BioProject, PRJNA796325; and BioSample, SAMN24847855.
